# Cardiac Gated Computed Tomography Angiography Discloses a Correlation Between the Volumes of All Four Cardiac Chambers and Heart Rate in Men But Not in Women

**DOI:** 10.1089/whr.2020.0052

**Published:** 2020-09-24

**Authors:** Tamar Shalmon, Yaron Arbel, Yoav Granot, Tomer Ziv-Baran, Ehud Chorin, Haim Shmilovich, Ofer Havakuk, Shlomo Berliner, Montserrat Carrillo Estrada, Galit Aviram

**Affiliations:** ^1^ Department of Radiology, Tel Aviv Medical Center, Tel Aviv, Affiliated to Sackler School of Medicine, Tel Aviv University, Tel Aviv, Israel.; ^2^ Department of Cardiology, and Tel Aviv Medical Center, Tel Aviv, Affiliated to Sackler School of Medicine, Tel Aviv University, Tel Aviv, Israel.; ^3^ Department of Internal Medicine E, Tel Aviv Medical Center, Tel Aviv, Affiliated to Sackler School of Medicine, Tel Aviv University, Tel Aviv, Israel.; ^4^ Department of Epidemiology and Preventive Medicine, School of Public Health, Sackler Faculty of Medicine, Tel Aviv University, Tel Aviv, Israel.; ^5^ Cardiac Intensive Care Unit, Cardiology Hospital, Centro Medico Nacional Siglo XXI, IMSS, Mexico City, Mexico.

**Keywords:** gender, cardiac-gated CT angiography, heart rate

## Abstract

***Background:*** Currently, normal values of the cardiac chambers' volumes are adjusted only for gender and body surface area (BSA). We aim to investigate the association between the heart rate and the volume of each of the four cardiac chambers using cardiac-gated computed tomography angiography (CCTA).

***Methods:*** A total of 350 consecutive patients without known cardiac diseases or significant (>50%) stenosis undergoing CCTA between January 2009 and June 2014 for suspected coronary artery disease were included. Cardiac chamber volumes adjusted to BSA were calculated using automated model-based segmentation analysis software of the CCTA data and correlated with patients' mean heart rate during the scan.

***Results:*** There were 240 men and 110 women, median interquartile range age was 55 years (47–61). Women were older 59.0 years (53.7–64) versus 52.0 years (45.0–59.0), had higher prevalence of hyperlipidemia, diabetes mellitus, anemia, and hypothyroidism, and higher median heart rates 64.0 (59.7–66.0) versus 60.0 (55.0–65.0) (*p* < 0.001). Men had a negative correlation between the volume of each cardiac chamber and the heart rate [*r*_age_adj_ = (−0.4)–(−0.27), *p* < 0.001 for all], whereas such a correlation was not found in women. The multivariate analysis showed that a decrease of five beats per minute was associated with an increase of 4%–5% in volume of each chamber in men. There was no such association among females.

***Conclusions:*** Lower heart rate is associated with an increase of each cardiac chamber volume by CCTA in men. This association is not found in women. More extensive studies are required to further elaborate on these gender differences.

## Background

Accurate evaluation of the heart volume can contribute to the diagnosis of heart pathologies and carries a prognostic value. Volumetric measurements of both ventricles and atria have become part of the routine in cardiac imaging. Currently, the assessment of the volumes of the cardiac chambers, in every modality, including echocardiography as well as cardiac magnetic resonance (CMR), is adjusted to gender and body surface area (BSA) only.^1^

Previous studies, mostly done using echocardiography, showed a correlation between the left ventricle (LV) size and the heart rate,^2,3^ in which patients with reduced LV size had faster heart rates.^4^ This can be explained by cardiac physiology: shorter duration of the LV filling phase with increasing heart rate leads to a decrease in the end-diastole volume (EDV) of the ventricle. None of the mentioned studies, however, investigated the correlation between heart rate and the alteration in each of the four cardiac chamber volumes according to gender.

During the past few years, a computed tomography (CT)-based 4-chamber volumetric analysis software (4CVA) was applied to automatically assess the cardiac chambers' volumes in patients who underwent cardiac-gated CT angiography (CCTA).^5^ One of the advantages of 4CVA technology is its capability to provide the volumes of all four cardiac chambers automatically and simultaneously. Thus, this study aimed to assess the relationship between the volumes of the four cardiac chambers based on the analysis of CCTA data and cardiac heart rate according to gender. We believe that the establishment of the relationship between heart rate and each cardiac chamber volume may contribute to refining the definitions of the normal volume of each chamber by additional adjustment to the heart rate.

## Methods

### Study design and patient selection

The data in this investigation constitute a part of the Tel Aviv Prospective Angiographic Survey (TAPAS). The TAPAS is a prospective single-center registry that enrolls all patients undergoing any cardiac test (angiogram, echocardiography, CCTA) at the Tel Aviv Medical Center.^6,7^ In this cross-sectional study, all consecutive ambulatory patients who underwent CCTA for the assessment of coronary artery disease (CAD) from January 2009 to June 2014 were included. Patients with known cardiac disease, that is, congestive heart failure, atrial fibrillation, and other arrhythmias, have been excluded. The medical history of each patient was documented through individual questionnaires and collected into a dedicated database. All the enrollees signed a written informed consent for participation in the study, which was approved by the institutional ethics committee.

Patients with data on their BSA and accurate cardiac chamber volumetric segmentation *via* 4CVA were included. Patients with evidence of significant CAD (≥50% stenosis in one or more coronary arteries), according to the present CCTA, were excluded.

### CT acquisition

CCTA was performed with a 128 × 2 × 0.625 mm or 64 × 0.625 mm detector rows scanner (ICT 256 or Brilliance 64; Philips Healthcare, Cleveland, OH) using our routine coronary arteries assessment protocol. In brief, an average of 70 to 90 mL of iodinated contrast material at a concentration of 400 mg iodine per mL (Iomeron; Bracco, Milano, Italy) and rates of 5 to 6 mL/sec were injected and timed using an automated bolus-tracking technique, placed at the descending aorta. To permit visualization of the right heart, we use three injection phases: first is the phase of contrast only (55–70 mL), second is the phase of mixed 50% contrast and 50% saline (total of 30–40 mL), and the third phase of 40 mL of saline flush. We used a prospective (step and shoot) mode scan for every patient with a heart rate <70 bpm or retrospective gating with dose modulation in patients with higher heart rates. During image acquisition, all patients were in sinus rhythm. Data were reconstructed at a slice thickness of 0.8 mm with an increment of 0.4 mm.

Oral β-blockers (metoprolol 50 mg) were given to patients who were not pretreated with β-blockers (*n* = 175) in case of a heart rate >60 bpm.

### Coronary artery and volumetric analysis of the cardiac chambers

**FIG. 1.**
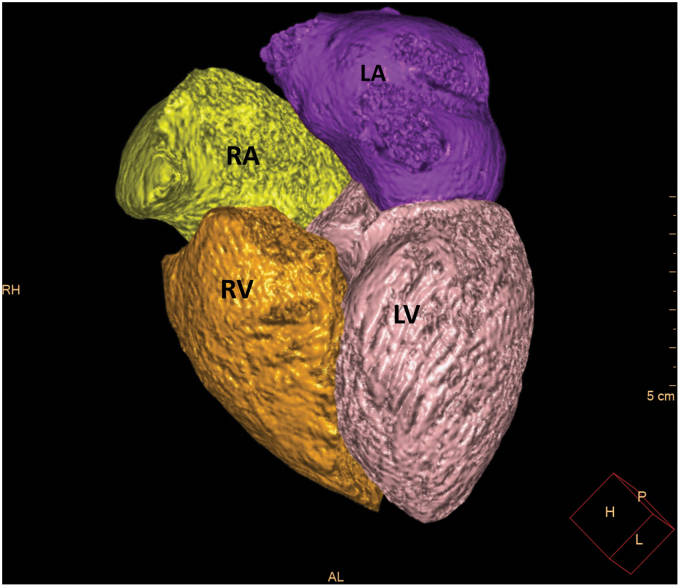
The output of the 4CVA for the horizontal long axis. Color code: LA, purple; LV, pink; RA, yellow; RV, orange. 4CVA, 4-chamber volumetric analysis software; LA, left atrium; LV, left ventricle; RA, right atrium; RV, right ventricle.

**FIG. 2.**
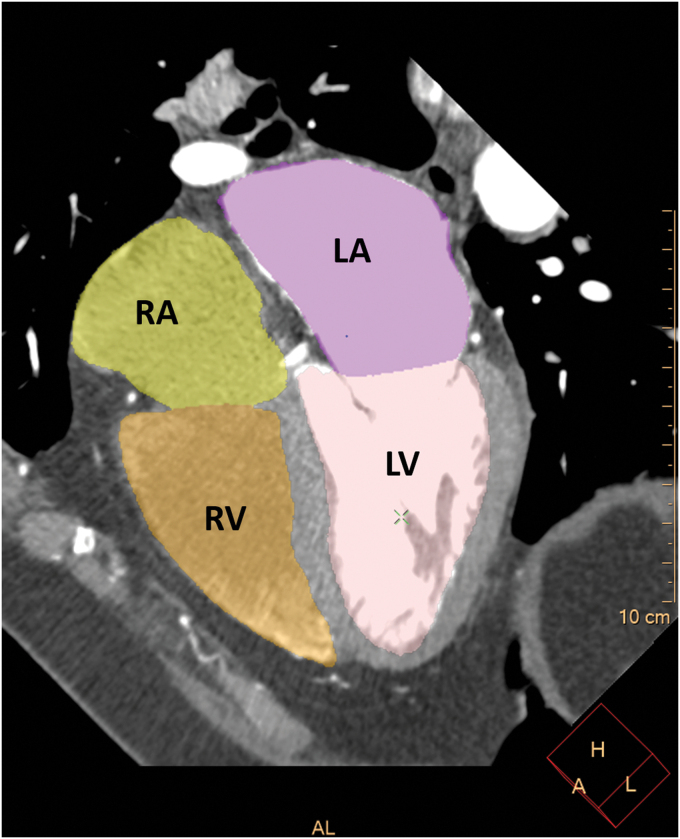
The output of the 4CVA for the volumetric model of the four cardiac chambers. Color code: LA, purple; LV, pink; RA, yellow; RV, orange.

**FIG. 3.**
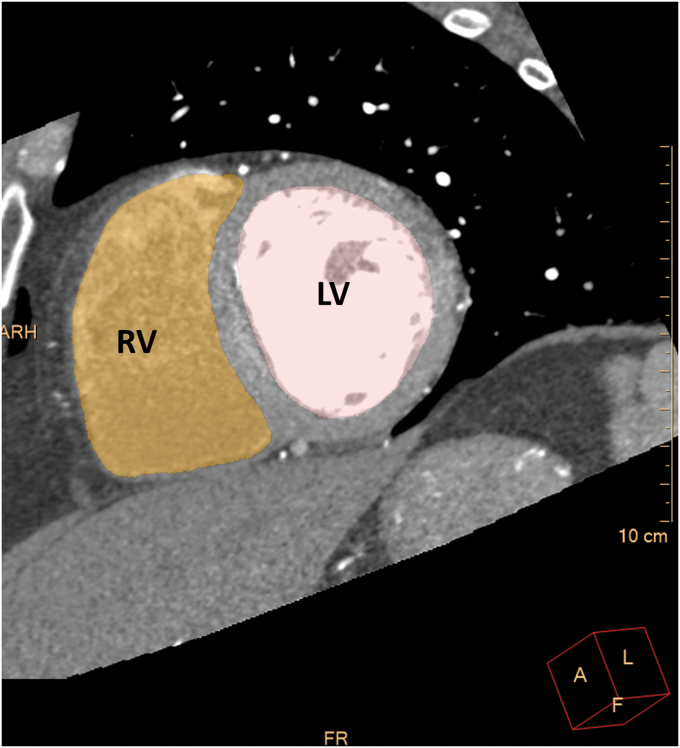
The output of the 4CVA for the short axis oblique reconstruction. Color code: LV, pink; RV, orange.

Automated volumetric measurements of the right ventricle (RV), right atrium (RA), LV, and the left atrium (LA) of the CCTA scans were obtained using fully automatic software (Comprehensive Cardiac Analysis, Extended Brilliance Workspace, Version 4.5 or Intelli-Space, Portal Version 6; Philips Healthcare). The software adapts an anatomical model of the heart chambers to the CT image volume.^8–10^ The output consists of a three-dimensional graphics display of the heart segmented into its main structures (Figs. 1–3). The volume of each cardiac chamber was automatically calculated as the product of a single voxel volume, and the sum of all voxels included in it. For the LV volume, LV myocardium was automatically excluded, and for LA volume, the pulmonary veins and the LA appendage were automatically excluded. For the RV, the area under pulmonary valve leaflet was included, and for the RA, the right atrial appendage was included, and the inferior vena cava was excluded. The software allows the relevant segmentation structure to be color coded and viewed simultaneously in both three dimension and two dimension images and superimposed on the reference image in the axial, coronal, sagittal, or cardiac views (short axis, long vertical axis, and long horizontal axis). For validation of segmentation correctness, each structure was inspected visually on the reference images for conformity to the imaged cardiac anatomy. In cases wherein the automatic segmentation was visually assessed as incorrect, the patient was excluded from the study.

The volumes of the four cardiac chambers were calculated at mid-diastole, which occurs at 75% or 78% of the cardiac cycle. All volumetric measurements were divided by the BSA and reported as mL/m^2^.

Also, using dedicated coronary analysis software of the same workspace unit, the coronary arteries were evaluated for the presence of CAD using the phase that had the least motion artifacts. Obstructive CAD was defined as evidence of ≥50% reduction of the diameter in one or more of the coronary arteries on CCTA.

### Statistical analysis

Categorical variables were expressed as numbers and percentages. The distribution of continuous variables was assessed using a histogram and Q–Q plot. Normally distributed continuous variables were described using mean and standard deviation, and non-normally distributed continuous variables were expressed using median and interquartile range (IQR). To account for the right-skewed distribution of chamber volumes, we used natural logarithm transformation in all statistical analyses. Categorical variables were compared using the chi-square test or Fisher's exact test, and continuous variables were compared using independent samples *t*-test or Mann–Whitney test, as appropriate. Associations between continuous variables were assessed by Pearson product–moment correlation coefficient or Spearman's rank correlation coefficient, as appropriate. Association between each chamber volume and pulse while controlling for the effect of age was assessed using partial correlation. Univariate and multivariate linear regressions were used to evaluate the percentage change in chamber volume for a five-beat change in pulse. The covariates included in the multivariate regression were age, gender, and variables with *p* < 0.2 in the univariate analysis. All linear regressions were evaluated to meet the regression assumptions: linear relationship and homoscedasticity using scatter plot, normality of the errors using a histogram and Q–Q plot, and no or little multicollinearity using variance inflation factor (VIF <5 was considered as no or little multicollinearity). A two-tailed *p* < 0.05 was considered statistically significant. Analyses were performed with SPSS (IBM Corp. Released in 2013. IBM SPSS Statistics for Windows, Version 22.0. Armonk, NY: IBM Corp.).

## Results

### Patient characteristics

Out of 480 patients referred for elective CCTA for suspected CAD, 130 patients (27%) were excluded due to known cardiac disease or significant (>50%) coronary stenosis on their CCTA, or inaccurate segmentation. Thus, the final cohort included 350 patients without a significant CAD, median age 55 years (IQR, 47–61 years), 240 men (68%).

Women were older with a median age of 59 years (IQR, 53.7–64 years) compared with men, 52 years (IQR, 45–59 years), (*p* < 0.001). Women had a higher prevalence of comorbidities, including hyperlipidemia (*p* = 0.037), diabetes mellitus (*p* < 0.001), anemia (*p* = 0.011), and hypothyroidism (*p* < 0.001). Women had also higher median heart rates 64.0 (59.7–66.0) than men 60.0 (55.0–65.0, *p* < 0.001). Other baseline characteristics are given in Table 1. Cardiac chamber volumes, calculated at mid-diastole, and adjusted to BSA, are given in Table 2. Although median LV and RA volumes adjusted to BSA were not different between genders, median LA volume was higher in women, and median RV volume was higher in men (*p* < 0.001).

**Table 1.** Baseline Characteristics of Patients According to Gender

N	All participants	Men	Women	p
	350	240	110	
Heart rate	60 (55–65)	60.0 (55–65)	64.0 (59.7–66.0)	<0.001
Age (years)	55 (47–61)	52.0 (45–59)	59.0 (53.7–64)	<0.001
Hypertension (%)	119 (36.6)	78 (34.7)	41 (41)	0.274
Diabetes mellites (%)	42 (12.9)	18 (8)	24 (24)	<0.001
Hyperlipidemia (%)	108 (41.1)	71 (37.2)	37 (51.4)	<0.037
Overweight (%)	90 (36.0)	62 (34.6)	28 (39.4)	0.476
COPD (%)	21 (6.6)	13 (6)	8 (7.9)	0.512
Hypothyroid (%)	14 (5.1)	3 (1.5)	11 (14.9)	<0.001
Hyperthyroid (%)	4 (1.5)	2 (1)	2 (3.0)	0.263
Anemia (%)	31 (8.9)	15 (6.3)	16 (14.5)	0.011
Current smoking (%)	74 (22.7)	56 (24.6)	18 (18.4)	0.444
β Blocker (%)	175 (50)	112 (46.7)	63 (57.3)	0.065

Values are presented as a number of patients, median (interquartile range), or percentages. *P*-value.

COPD, chronic obstructive pulmonary disease.

**Table 2.** Median Cardiac Chamber Volumes Adjusted to Body Surface Area with Respect to Gender

N	All cohort	Men	Women	p
	350	240	110	
LV volume/BSA (mL/sqm)	59.4 (52.4–69.2)	60.7 (52.0–70.5)	57.9 (52.7–63.1)	0.348
LA volume/BSA (mL/sqm)	36.0 (30.6–43.1)	34.3 (29.4–42.8)	38.1 (33.2–43.6)	<0.001
RV volume/BSA (mL/sqm)	73.1 (64.7–83.3)	78.1 (69.8–87.9)	65.0 (57.1–71.3)	<0.001
RA volume/BSA (mL/sqm)	41.0 (35.1–47.3)	41.3 (35.0–48.0)	40.4 (35.4–45.9)	0.599

The measures are median and interquartile range (IQR).

BSA, body surface area; LA, left atrium; LV, left venticle; RA, right atrium; RV, right ventricle.

Table 3 gives the results of the volumetric analysis: men had a negative correlation between the volume of each cardiac chamber and the heart rate (*r*_age_adj_ = (−0.4)–(−0.27), *p* < 0.001 for all cardiac chambers), whereas there was no correlation in women.

**Table 3.** Correlation Between the Volume of Each Cardiac Chamber and Heart Rate, Age, and Heart Rate Adjusted

Volume/BSA (mL/sqm)	Men			Women		
	Heart rate	Age	Heart rate (controlling for age)	Heart rate	Age	Heart rate (controlling for age)
LV: r	−0.26	−0.26	−0.27	−0.13	−0.27	−0.16
*p*	<0.001	<0.001	<0.001	0.194	0.005	0.107
LA: r	−0.29	0.34	−0.30	0.01	0.42	0.06
*p*	<0.001	<0.001	<0.001	0.905	<0.001	0.569
RV: r	−0.37	−0.31	−0.4	−0.05	−0.34	−0.09
*p*	<0.001	<0.001	<0.001	0.611	<0.001	0.38
RA: r	−0.29	0.22	−0.3	0.01	0.4	0.05
*p*	<0.001	0.001	<0.001	0.904	<0.001	0.59

**FIG. 4.**
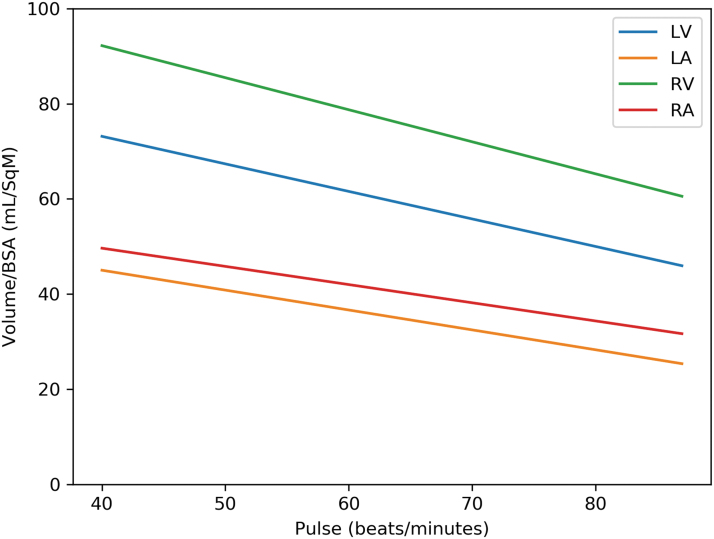
Cardiac chamber volumes versus heart rate in men.

**FIG. 5.**
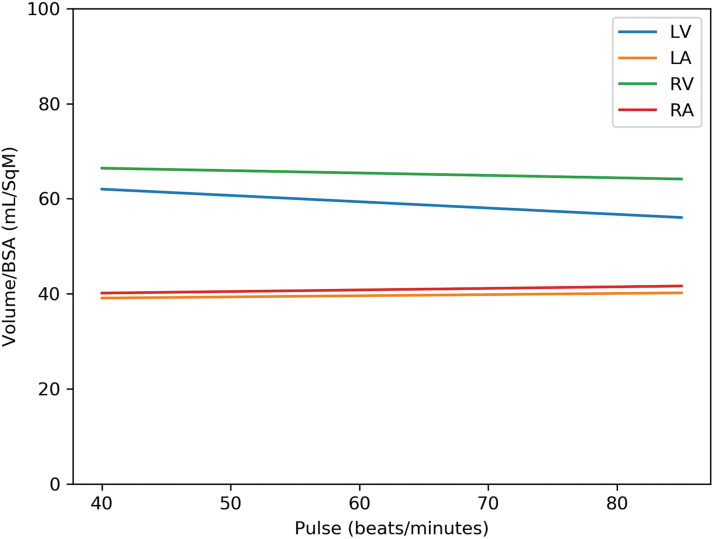
Cardiac chamber volumes versus heart rate in women.

Table 4 gives the multivariate analysis adjusted for age and variables, which had *p* ≤ 0.2 in univariate analysis. Hypertension, diabetes mellitus, hyperlipidemia, hypothyroidism, overweight, chronic obstructive pulmonary disease, anemia, smoking, and β-blocker were considered as potential confounders for inclusion in multivariate analysis. Among men, each chamber volume indexed to BSA was negatively associated with the heart rate; for every increase of 5 beats/min of the heart rate, there is a decrease in heart chambers volume of 4% in the volume of the LV/BSA; 4.9% in the volume of the LA/BSA; 4.5% in the volume of the RV/BSA; and 4.9% in the volume of the RA/BSA (Fig. 4). There was no such association among women (Fig. 5).

**Table 4.** Crude and Adjusted Percentage of Change in Volume/BSA for Five Beats/Min Changed in Heart Rate

Chamber	Type of analysis	Men		Women	
		Percentage change for five beats pulse (95% CI)	p	Percentage change for five beats pulse (95% CI)	p
LV	Crude	−4.81 (−7.03, −2.53)	<0.001	−1.19 (−2.97, 0.62)	0.194
	Adjusted	−4.0 (−6.18, −1.72)	<0.001	−1.52 (−3.18, 0.16)	0.076
LA	Crude	−5.41 (−7.57, −3.2)	<0.001	0.14 (−2.18, 2.52)	0.905
	Adjusted	−4.9 (−6.92, −2.82)	<0.001	0.88 (−1.2, 3.01)	0.407
RV	Crude	−4.38 (−5.76, −2.99)	<0.001	−0.42 (−2.05, 1.23)	0.611
	Adjusted	−4.5 (−5.81, −3.11)	<0.001	−0.46 (−1.95, 1.06)	0.550
RA	Crude	−4.89 (−6.89, −2.84)	<0.001	0.14 (−2.17, 2.51)	0.904
	Adjusted	−4.9 (−6.89, −2.91)	<0.001	0.76 (−1.38, 2.94)	0.488

Adjusted to age and variables, which had *p* < 0.2 in univariate analysis. Hypertension, diabetes mellitus, hyperlipidemia, overweight, chronic obstructive pulmonary disease, anemia, smoking, and β-blocker were considered as potential confounders for inclusion in multivariate analysis.

## Discussion

The ability of alterations in heart rate to influence LV volumes and ejection fraction has been well recognized for many years.^2–4,11^ Previous studies, mainly done using echocardiography, showed a correlation between the LV volumes and the heart rate, in which patients with faster heart rates had reduced LV EDV and end-systolic volume (ESV).^4,11^ However, according to the best of our knowledge, none of the previous studies investigated the relationship between heart rate and the alteration in all four cardiac chamber volumes simultaneously at rest using CCTA. In addition, despite the presence of observations regarding the effect of alterations in heart rate on LV volumes, these studies were done mainly among men and did not provide viable information on the effect of heart rate increase on heart chamber volumes among women.^2–4,11–18^

CCTA is nowadays widely used for the assessment of CAD.^19^ The same imaging study may provide in addition data on the volumes of each of the cardiac chambers, including the LA, RV, and RA, which can be valuable. This software was trained to identify the various cardiac compartments based on a prelearned anatomical model, thus enabling efficient workflow by automated cardiac chamber volume calculation. The output of the automated calculations was compared with the results from labor-intensive manual segmentation and found to be highly reproducible and accurate.^5^ Normal values for mid-diastolic LV and LA volume have been proposed previously by Walker et al.,^20^ showing a good correlation between mid-diastolic volumes compared with the maximum volumes of these chambers. Other methods like echocardiography have limited ability for the assessment of the right heart chambers due to the complex shape of the RV, and restrictions of the field of view and the limited acoustic window.

Investigations of the effect of increased heart rate on LV volume during physical exercise as opposed to resting are numerous but are mostly performed on men only.^12–18,21–23^ This study, which contains a relatively large number of women, shows that although among men there is a significant negative correlation between heart rate and cardiac chamber volumes, such a correlation does not present among women. It is well known from the literature that gender differences exist in normal heart structure and function.^24–28^ Salton et al.^24^ defined gender-specific normal LV CMR reference values in 142 subjects free of clinically apparent cardiovascular disease or clinical history of hypertension (63 men, 79 women; age 57 ± 9 years) and Lorenz et al.^25^ reported normal LV CMR values in relatively young 75 healthy subjects (47 men, 28 women; age 28 ± 9 years). Both investigators reported higher LV volume in men, before and after adjustment for height and BSA. Other studies supported those results and showed that men have higher EDV and ESV, greater mass, and lower LV ejection fraction than women.^26,27^ Similar findings were observed by Robbers-Visser et al.^28^ in the pediatric population (60 healthy children, 30 boys; aged 8–17 years), supporting the hypothesis that cardiac volumes are related to gender more than to other factors such as body size.

In this investigation, there was no significant difference in the LV volume between men and women, and women had larger LA. A possible explanation for this could be that in our cohort study population, women were older with a higher prevalence of comorbidities, such as diabetes mellitus, than men. It has been shown in previous studies that diastolic dysfunction is one of the findings in diabetic cardiomyopathy,^29^ which can explain the larger LA volume in women.^30^ Smaller LV EDV indexed and LV ESV indexed have been found to be early changes of the cardiac structure in patients with diabetes with the use of cardiac MRI, without significant changes in LV ejection fraction measures.^31^ Another large prospective study found that the chronic influence of diabetes may manifest with LV dilatation (and eventually a decline in LV systolic function).^32^ However, patients included in this study referred for coronary CTA; therefore, they do not have echocardiography, and the diastolic function was not evaluated for the patients included.

Regarding the right heart, similar to our findings, previous studies have shown that women have lower RV volumes and higher RV ejection fraction than men.^33–35^

In this study, men had a negative correlation between the volume of each of the four cardiac chambers and the heart rate, whereas such a correlation was not found in women. Extensive literature demonstrating gender differences in cardiovascular disease exists,^36–39^ but to the best of our knowledge, gender difference with regard to the relationships between the cardiac volumes and the heart rate was not reported. Some studies showed that cardiac contractility is greater in healthy women than in age-matched men,^40^ and hormone replacement therapy withdrawal in women decreases contractility.^41^ Gender affects cardiac electrophysiological properties: women have a faster heart rate that men, estrogen increases the number of β-adrenergic receptors in the myocardium. It has been shown before that the sinus cycle length, i.e. QRS duration and HV interval, are longer in men.^42^ Hormonal influences could explain these findings.^43–45^

Another possible factor that may have contributed to the high gender-related difference of the cardiac chambers' volumes in association with the individual's heart rate in our research might be related to the difference in the age and background diseases between the subjects' gender, since women were older with more comorbidities, which comprises one of the major limitations of this study. Only 2 (3%) of the women in our study had hyperthyroidism, and 11(15%) of the women had hypothyroidism. Hypothyroidism can have effects on heart function such as a reduction in heart rate, decrease in myocardium contractility, and alteration in the diastolic function by slowing the isovolumic relaxation phase.^46^

Another drawback is the use of β-blockers. Since the purpose of the CCTA was the evaluation of CAD, β-blockers were used in patients with prescan heart rate >60 bpm, to obtain the sharpest images of the coronary arteries. By using β-blockers in half of the patients, we artificially changed, that is, slowed down, the normal physiological heart rate of those patients. β-Blockers can increase LV ejection fraction by reducing ESV and optimizing ventricular filling.^47^ Differences in gender response to β-blockers have also been reported, women have higher peak plasma levels of some β-blockers than men, because of enhanced absorption. This could lead to a greater reduction in heart rate in women.^48^ Since CT technology is still associated with radiation exposure, there is, however, no legitimate use of large series of volunteers for the purpose of research only; thus, we consequently had to take advantage of the CT scans that were done for clinical purposes. Nevertheless, the obtained volumes were correlated to the actual heart rate during CT acquisition itself, even if it was slowed down by the premedication, and thus they still reflect the actual association between the heart rate and the volume of each of the four cardiac chambers. In addition, the rate of β blocker use was similar between genders. Thus they are likely not influencing the finding related to gender differences.

## Conclusions

In summary, using the 4CVA technology, this study shows that among men, for every increase of 5 beats/min of the heart rate, there is a decrease in volume of each of the heart chambers in 4%–5%, whereas in women such an association does not exist. Thus, for establishing accurate normal limit values of heart chamber volumes, additional adjustment to heart rate in men should be considered. Since this association was not found in women, more extensive studies are required to elaborate on these gender differences further.

## Abbreviations Used

4CVA: 4-chamber volumetric analysis software
BSA: body surface area
CAD: coronary artery disease
CCTA: cardiac-gated computed tomography angiography
CMR: cardiac magnetic resonance
CT: computed tomography
EDV: end-diastole volume
ESV: end-systole volume
IQR: interquartile range
LA: left atrium
LV: left ventricle
RA: right atrium
RV: right ventricle
TAPAS: Tel Aviv Prospective Angiographic Survey
VIF: variance inflation factor

## References

[1] Lang RM, Badano LP, Mor-Avi V, et al. Recommendations for cardiac chamber quantification by echocardiography in adults: An update from the American Society of Echocardiography and the European Association of Cardiovascular Imaging. Eur Heart J Cardiovasc Imaging 2015;16:233–271 25712077 10.1093/ehjci/jev014

[2] Glick G, Sonnenblick EH, Braunwald E Myocardial force-velocity relations studied in intact unanesthetized man. J Clin Invest 1965;44:978–988 14322032 10.1172/JCI105215 PMC292578

[3] Hirshleifer J, Crawford M, O'Rourke RA, Karliner JS Influence of acute alterations in heart rate and systemic arterial pressure on echocardiographic measures of left ventricular perfornmance in normal human subjects. Circulation 1975;52:835–841 1175264 10.1161/01.cir.52.5.835

[4] DeMaria AN, Neumann A, Schubart PJ, Lee G, Mason DT Systematic correlation of cardiac chamber size and ventricular performance determined with echocardiography and alterations in heart rate in normal persons. Am J Cardiol 1979;43:1–9 758757 10.1016/0002-9149(79)90036-5

[5] Mao SS, Li D, Vembar M, et al. Model-based automatic segmentation algorithm accurately assesses the whole cardiac volumetric parameters in patients with cardiac CT angiography: A validation study for evaluating the accuracy of the workstation software and establishing the reference val. Acad Radiol 2014;21:639–647 24703477 10.1016/j.acra.2014.01.010

[6] Arbel Y, Zlotnik M, Halkin A, et al. Admission glucose, fasting glucose, HbA1c levels and the SYNTAX score in non-diabetic patients undergoing coronary angiography. Clin Res Cardiol 2014;103:223–227 24271460 10.1007/s00392-013-0641-7

[7] Arbel Y, Shenhar-Tsarfaty S, Waiskopf N, et al. Decline in serum cholinesterase activities predicts 2-year major adverse cardiac events. Mol Med 2014;20:38–45 24395570 10.2119/molmed.2013.00139 PMC3951463

[8] Abadi S, Roguin A, Engel A, Lessick J Feasibility of automatic assessment of four-chamber cardiac function with MDCT: Initial clinical application and validation. Eur J Radiol 2010;74:175–181 19261417 10.1016/j.ejrad.2009.01.035

[9] Ecabert O, Peters J, Schramm H, et al. Automatic model-based segmentation of the heart in CT images. IEEE Trans Med Imaging 2008;27:1189–1201 18753041 10.1109/TMI.2008.918330

[10] Lorenz C, von Berg J A comprehensive shape model of the heart. Med Image Anal 2006;10:657–670 16709463 10.1016/j.media.2006.03.004

[11] Erbel R, Schweizer P, Krebs W, Langen H-J, Meyer J, Effert S Effects of heart rate changes on left ventricular volume and ejection fraction: A 2-dimensional echocardiographic study. Am J Cardiol 1984;53:590–597 6695789 10.1016/0002-9149(84)90036-5

[12] Bar-Shlomo BZ, Druck MN, Morch JE, et al. Left ventricular function in trained and untrained healthy subjects. Circulation 1982;65:484–488 7055870 10.1161/01.cir.65.3.484

[13] Crawford MH, White DH, Amon KW Echocardiographic evaluation of left ventricular size and performance during handgrip and supine and upright bicycle exercise. Circulation 1979;59:1188–1196 436212 10.1161/01.cir.59.6.1188

[14] Fagard R, Van Den Broeke C, Amery A Left ventricular dynamics during exercise in elite marathon runners. J Am Coll Cardiol 1989;14:112–118 2738255 10.1016/0735-1097(89)90060-0

[15] Henriksen E, Sundstedt M, Hedberg P Left ventricular end-diastolic geometrical adjustments during exercise in endurance athletes. Clin Physiol Funct Imaging 2008;28:76–80 18076659 10.1111/j.1475-097X.2007.00768.x

[16] Rerych SK, Scholz PM, Sabiston DC Jr, Jones RH Effects of exercise training on left ventricular function in normal subjects: A longitudinal study by radionuclide angiography. Am J Cardiol 1980;45:244–252 7355734 10.1016/0002-9149(80)90642-6

[17] Sundstedt M, Hedberg P, Jonason T, Ringqvist I, Brodin L, Henriksen E Left ventricular volumes during exercise in endurance athletes assessed by contrast echocardiography. Acta Physiol Scand 2004;182:45–51 15329056 10.1111/j.1365-201X.2004.01304.x

[18] Steding-Ehrenborg K, Jablonowski R, Arvidsson PM, Carlsson M, Saltin B, Arheden H Moderate intensity supine exercise causes decreased cardiac volumes and increased outer volume variations: A cardiovascular magnetic resonance study. J Cardiovasc Magn Reson 2013;15:96 24156367 10.1186/1532-429X-15-96 PMC4015552

[19] Montalescot G, Sechtem U, Achenbach S, et al. 2013 ESC guidelines on the management of stable coronary artery disease: The Task Force on the management of stable coronary artery disease of the European Society of Cardiology. Eur Heart J 2013;34:2949–3003 23996286 10.1093/eurheartj/eht296

[20] Walker JR, Abadi S, Solomonica A, et al. Left-sided cardiac chamber evaluation using single-phase mid-diastolic coronary computed tomography angiography: Derivation of normal values and comparison with conventional end-diastolic and end-systolic phases. Eur Radiol 2016;26:3626–3634 26809292 10.1007/s00330-016-4211-z

[21] Steding K, Engblom H, Buhre T, et al. Relation between cardiac dimensions and peak oxygen uptake. J Cardiovasc Magn Reson 2010;12:8 20122149 10.1186/1532-429X-12-8 PMC2825210

[22] Widmaier EP, Raff H, Strang KT, Vander AJ Vander's human physiology: The mechanisms of body function. Boston: McGraw-Hill Higher Education, 2008

[23] Stein RA, Michielli D, Diamond J, Horwitz B, Krasnow N The cardiac response to exercise training: Echocardiographic analysis at rest and during exercise. Am J Cardiol 1980;46:219–225 7405835 10.1016/0002-9149(80)90061-2

[24] Salton CJ, Chuang ML, O'Donnell CJ, et al. Gender differences and normal left ventricular anatomy in an adult population free of hypertension: A cardiovascular magnetic resonance study of the Framingham Heart Study Offspring cohort. J Am Coll Cardiol 2002;39:1055–1060 11897450 10.1016/s0735-1097(02)01712-6

[25] Lorenz CH, Walker ES, Morgan VL, Klein SS, Graham TP Normal human right and left ventricular mass, systolic function, and gender differences by cine magnetic resonance imaging. J Cardiovasc Magn Reson 1999;1:7–21 11550343 10.3109/10976649909080829

[26] Maceira AM, Prasad SK, Khan M, Pennell DJ Reference right ventricular systolic and diastolic function normalized to age, gender and body surface area from steady-state free precession cardiovascular magnetic resonance. Eur Heart J 2006;27:2879–2888 17088316 10.1093/eurheartj/ehl336

[27] Hudsmith LE, Petersen SE, Francis JM, Robson MD, Neubauer S Normal human left and right ventricular and left atrial dimensions using steady state free precession magnetic resonance imaging. J Cardiovasc Magn Reson 2005;7:775–782 16353438 10.1080/10976640500295516

[28] Robbers-Visser D, Boersma E, Helbing WA Normal biventricular function, volumes, and mass in children aged 8 to 17 years. J Magn Reson Imaging An Off J Int Soc Magn Reson Med 2009;29:552–559 10.1002/jmri.21662 19243036

[29] Poirier P, Bogaty P, Garneau C, Marois L, Dumesnil J-G Diastolic dysfunction in normotensive men with well-controlled type 2 diabetes: Importance of maneuvers in echocardiographic screening for preclinical diabetic cardiomyopathy. Diabetes Care 2001;24:5–10 11194240 10.2337/diacare.24.1.5

[30] Tsang TSM, Barnes ME, Gersh BJ, Bailey KR, Seward JB Left atrial volume as a morphophysiologic expression of left ventricular diastolic dysfunction and relation to cardiovascular risk burden. Am J Cardiol 2002;90:1284–1289 12480035 10.1016/s0002-9149(02)02864-3

[31] Jensen MT, Fung K, Aung N, et al. Changes in cardiac morphology and function in individuals with diabetes mellitus: The UK Biobank cardiovascular magnetic resonance substudy. Circ Cardiovasc Imaging 2019;12:e009476 31522551 10.1161/CIRCIMAGING.119.009476 PMC7099857

[32] Markus MRP, Stritzke J, Wellmann J, et al. Implications of prevalent and incident diabetes mellitus on left ventricular geometry and function in the ageing heart: The MONICA/KORA Augsburg cohort study. Nutr Metab Cardiovasc Dis 2011;21:189–196 19939647 10.1016/j.numecd.2009.09.005

[33] Tamborini G, Marsan NA, Gripari P, et al. Reference values for right ventricular volumes and ejection fraction with real-time three-dimensional echocardiography: Evaluation in a large series of normal subjects. J Am Soc Echocardiogr 2010;23:109–115 20152691 10.1016/j.echo.2009.11.026

[34] Tandri H, Daya SK, Nasir K, et al. Normal reference values for the adult right ventricle by magnetic resonance imaging. Am J Cardiol 2006;98:1660–1664 17145230 10.1016/j.amjcard.2006.07.049

[35] Kawut SM, Lima JAC, Barr RG, et al. Sex and race differences in right ventricular structure and function: The Multi-Ethnic Study of Atherosclerosis–Right Ventricle Study. Circulation 2011;123:2542–2551 21646505 10.1161/CIRCULATIONAHA.110.985515 PMC3111939

[36] De Maria R, Gavazzi A, Recalcati F, Baroldi G, De Vita C, Camerini F Comparison of clinical findings in idiopathic dilated cardiomyopathy in women versus men. Am J Cardiol 1993;72:580–585 8362774 10.1016/0002-9149(93)90355-g

[37] Deswal A, Bozkurt B Comparison of morbidity in women versus men with heart failure and preserved ejection fraction. Am J Cardiol 2006;97:1228–1231 16616031 10.1016/j.amjcard.2005.11.042

[38] Douglas PS, Katz SE, Weinberg EO, Chen MH, Bishop SP, Lorell BH Hypertrophic remodeling: Gender differences in the early response to left ventricular pressure overload. J Am Coll Cardiol 1998;32:1118–1125 9768741 10.1016/s0735-1097(98)00347-7

[39] Ghali JK, Krause-Steinrauf HJ, Adams KF, et al. Gender differences in advanced heart failure: Insights from the BEST study. J Am Coll Cardiol 2003;42:2128–2134 14680739 10.1016/j.jacc.2003.05.012

[40] Mendelsohn ME, Karas RH Molecular and cellular basis of cardiovascular gender differences. Science 2005;308:1583–1587 15947175 10.1126/science.1112062

[41] Levy D, Garrison RJ, Savage DD, Kannel WB, Castelli WP Prognostic implications of echocardiographically determined left ventricular mass in the Framingham Heart Study. N Engl J Med 1990;322:1561–1566 2139921 10.1056/NEJM199005313222203

[42] Taneja T, Mahnert BW, Passman ROD, Goldberger J, Kadish A Effects of sex and age on electrocardiographic and cardiac electrophysiological properties in adults. Pacing Clin Electrophysiol 2001;24:16–21 11227963 10.1046/j.1460-9592.2001.00016.x

[43] Skavdahl M, Steenbergen C, Clark J, et al. Estrogen receptor-β mediates male-female differences in the development of pressure overload hypertrophy. Am J Physiol Circ Physiol 2005;288:H469–H476 10.1152/ajpheart.00723.2004 15374829

[44] Van Eickels M, Grohé C, Cleutjens JPM, Janssen BJ, Wellens HJJ, Doevendans PA 17β-Estradiol attenuates the development of pressure-overload hypertrophy. Circulation 2001;104:1419–1423 11560859 10.1161/hc3601.095577

[45] Mendelsohn ME, Karas RH The protective effects of estrogen on the cardiovascular system. N Engl J Med 1999;340:1801–1811 10362825 10.1056/NEJM199906103402306

[46] Klein I, Danzi S Thyroid disease and the heart. Circulation 2007;116:1725–1735 17923583 10.1161/CIRCULATIONAHA.106.678326

[47] Silke B Beta-blockade in CHF: Pathophysiological considerations. Eur Hear J Suppl 2006;8(suppl_C):C13–C18

[48] Tamargo J, Rosano G, Walther T, et al. Gender differences in the effects of cardiovascular drugs. Eur Hear J Cardiovasc Pharmacother 2017;3:163–182 10.1093/ehjcvp/pvw042 28329228

